# Effect of the depot medroxyprogesterone acetate injectable and levonorgestrel implant on HIV genital shedding: a randomized trial^☆^^☆☆^^★^^★★^

**DOI:** 10.1016/j.contraception.2018.05.001

**Published:** 2018-09

**Authors:** Lameck Chinula, Julie A.E. Nelson, Jeffrey Wiener, Jennifer H. Tang, Stacey Hurst, Gerald Tegha, Albans Msika, Sascha Ellington, Mina C. Hosseinipour, Ronald Mataya, Lisa B. Haddad, Athena P. Kourtis

**Affiliations:** a University of North Carolina at Chapel Hill, Department of Obstetrics & Gynecology, Chapel Hill, USA; b UNC Project-Malawi, Lilongwe, Malawi; c Malawi College of Medicine, Department of Obstetrics & Gynecology, Blantyre, Malawi; d University of North Carolina at Chapel Hill, Department of Microbiology and Immunology, Chapel Hill, USA; e US. Centers for Disease Control and Prevention, Division of Reproductive Health, USA; f University of North Carolina at Chapel Hill, Division of Infectious Diseases, Chapel Hill, USA; g Loma Linda School of Public Health, Loma Linda, USA; h Emory University, of Obstetrics & Gynecology, Atlanta, GA, USA

**Keywords:** HIV, Genital shedding, Progestin contraception, Levonorgestrel implant, Depot medroxyprogesterone acetate injectable

## Abstract

**Objectives:**

To assess the effect of the depot medroxyprogesterone acetate injectable (DMPA) and of the levonorgestrel (LNG) implant on genital HIV shedding among women receiving antiretroviral therapy (ART).

**Methods:**

We randomized HIV-infected Malawian women to either DMPA or LNG implant from May 2014 to April 2015. HIV RNA was measured in cervicovaginal lavage (CVL) fluid and TearFlo Strips (TFS), and HIV DNA was measured in cells collected by CVL. We compared the frequency and magnitude of HIV genital shedding before and for 6 months after initiation of contraception and between arms among women receiving ART. We also compared genital HIV RNA levels obtained by sample type (TFS versus CVL).

**Results:**

We analyzed data for 68 HIV-infected women receiving ART: 33 randomized to DMPA and 35 randomized to the LNG implant. Overall, HIV RNA was more often detectable and the quantity was higher on TFS compared with CVL. HIV DNA was detected very rarely in CVL cell samples (4 of 360 samples). The frequency of genital shedding and the genital HIV quantity did not increase after contraceptive initiation with either DMPA or LNG implant among women receiving ART.

**Conclusions:**

HIV-infected women receiving ART initiating contraception with either DMPA or LNG implant did not have any increase in genital HIV shedding during the first 6 months of contraceptive use. These findings are consistent with growing evidence that progestin contraception is not associated with increased HIV transmission risk from such women to their male partners. Consistent with other studies, genital HIV RNA detection was higher in TFS than in CVL fluid.

**Implications:**

In this randomized trial, neither DMPA nor the LNG implant, two of the most commonly used hormonal contraceptives among African women with HIV, was associated with increased genital HIV shedding in HIV-infected women receiving ART**.** These findings are reassuring and add to the currently limited information available for the highly effective contraceptive, LNG implant.

## 1 Introduction

Worldwide, there are over 16 million women living with human immunodeficiency virus type 1 (HIV) [1]. Use of effective contraception can help prevent unintended pregnancy and decrease mother-to-child transmission of HIV. Progestin-only contraceptives, such as the depot medroxyprogesterone acetate (DMPA) injectable and the levonorgestrel implant (LNG implant), are among the most commonly used and effective forms of modern contraception [2], [3]. However, hormonal contraceptives, particularly DMPA, have been linked in some studies with increased risk of HIV acquisition in uninfected women and increased risk of HIV transmission from infected women to partners [4], [5]. Given that heterosexual transmission is a common mode of HIV transmission, this concern about hormonal contraception is of great public health importance.

Plasma HIV RNA viral load is a strong predictor of heterosexual transmission, and antiretroviral therapy (ART) effectively decreases viral load in both the blood and the genital tract [6], [7]. However, HIV may remain present in the cervicovaginal secretions of patients receiving ART even if the individual has undetectable plasma viral load [8]. Genital tract HIV RNA viral load from cervical swab was shown to correlate significantly but imperfectly with plasma viral load (*r*=0.56) and to be an independent risk factor for HIV transmission [7]. Some studies have shown an association between hormonal contraceptive use and increased frequency of shedding of HIV-1 DNA but not RNA in the genital tract [8], [9], [10], [11], [12]. The association with HIV DNA has not been observed consistently in all studies [13], and the existing studies have several methodologic limitations and differences (such as type of genital sample tested and assay used) that challenge the interpretation and generalizability of their findings [14]. Additionally, there is limited information about this association in the context of ART, which is expected to have a major impact in preventing HIV transmission [15]. Therefore, the World Health Organization (WHO) encourages further studies on the impact of hormonal contraception on HIV acquisition and transmission [16].

Viral load in the genital tract can be measured in different specimens, such as cervical or vaginal swabs, cervical or vaginal wicks (Sno-Strips or TearFlo strips), and cervicovaginal lavage (CVL) fluid. Each of these specimen types has its advantages and disadvantages and may yield different results. The use of progestin is expected to induce changes in the thickness and other characteristics of cervical mucus, which can affect viral concentrations. HIV RNA viral loads from CVL are typically lower than other specimen types due to dilution of secretions during lavage [17], [18]. However, CVL samples more locations in the cervix and vagina as shown by HIV sequence differences between CVL and wicks [19].

We conducted a randomized clinical trial to assess the effect and compare the impact of two progestin-only contraceptives, DMPA and the LNG implant, on HIV shedding in the genital tract of HIV-infected women receiving ART [20]. We also compared genital HIV shedding and detection between TearFlo Strips (TFS) and CVL samples at each study visit to evaluate which sample type had higher detection rates.

## 2 Methods

### 2.1 Study design and enrollment

We recruited study participants at Bwaila Hospital, a large district hospital in Lilongwe, Malawi, by informing potentially eligible women about the study. Interested women provided informed consent for screening, completed an interview to confirm eligibility and underwent blood pressure measurement, urine pregnancy testing and HIV testing with both the Unigold™ and Determine® HIV rapid tests.

Inclusion criteria were: (1) age 18–45 years; (2) known HIV-infected status (as documented during screening); (3) self-report of at least two regular, monthly cycles (~21–35 days) in the 3 months preceding study enrollment; (4) self-report of not being on hormonal or intrauterine contraception for at least the preceding 6 months (if previously using DMPA, last injection must have been ≥6 months ago); (5) at least 6 months postpartum if recently delivered; (6) interested in initiating DMPA or the LNG implant; (7) willing to be randomized to receive either DMPA or the LNG implant; and (8) willing to wait 4–6 weeks after enrollment to receive the method and to use nonhormonal and nonintrauterine methods consistently during this period by self-report.

We excluded women who were pregnant at screening or desiring pregnancy within the next 12 months, had any medical contraindications to DMPA or LNG implant per the WHO medical eligibility criteria [21], or were newly diagnosed with HIV at screening with a known negative HIV test within the past 6 months. The reason for excluding known new HIV infections is that the plasma and mucosal HIV RNA viral loads are typically much higher in the acute setting and not representative of the status after the viral set point has been set.

We consented eligible women for enrollment and completed an interview that included questions on demographic, medical and reproductive health information. Visit 1 was completed that same day if they were within the first 14 days of their last menses (follicular phase) by self-report. Otherwise, they were scheduled for visit 1 to occur within the first 14 days of their next anticipated menstrual cycle. At visit 1, an interval history assessment and physical exam were completed. Participants with untreated visible genital ulcers or lesions at the initial pelvic examination were terminated from the study. Visit 2 was scheduled to occur during the luteal phase of the participant's cycle (from day 15 from the onset of her last menses until the start of her next menses). Visit 2 was completed in the same cycle that visit 1 was completed, unless the participant missed the visit in that cycle, in which case visit 2 would be rescheduled to the luteal phase of the subsequent cycle. Using permuted-block randomization, enrolled women were randomized to DMPA or the LNG implant and initiated on their randomized method at visit 3, which occurred during the first 7 days after the start of their next menses; we did not quick-start participants on contraception because we wanted to ensure that they had regular cycles every 21–35 days prior to randomization. Women were then scheduled to return on day 3 (visit 4), day 30 (visit 5), day 90 (visit 6) and day 180 (visit 7) after randomization.

The target sample size for randomized participants was 100 HIV-infected women; we estimated that approximately two thirds (67 women) would be receiving ART at the time of enrollment. Assuming a mean of 2.0 log_10_ cervical HIV RNA level prior to initiation of contraception [10], [22], we determined we would be able to detect a 0.51 log_10_ difference in mean HIV RNA after initiation of the contraceptive and a 0.50 log_10_ difference in mean viral shedding between the two randomized arms with a power of 80%, *α*=0.05 and a 10% loss-to-follow-up rate.

### 2.2 Specimen collection

At visits 1, 2, 4, 5, 6 and 7, we collected blood and genital samples (TFS and CVL). Collected blood was separated into plasma that was stored at −80°C. We collected TFS from each woman first by holding two strips in fornix for approximately 1 min and transferred them to a tube for frozen storage. CVL was collected by lavaging the cervix, vaginal walls and posterior fornix with 10 ml of phosphate-buffered saline and then aspirating the pooled fluid [23]. Collected CVL was centrifuged and the supernatant fluid stored at −80°C; pelleted CVL cells were stored as a dry pellet at −80°C. Collection of TFS and CVL was rescheduled if the woman had any vaginal bleeding at the time of the study visit, and if there were no days without bleeding during the study visit window, then the visit was not conducted and the sample was missed.

### 2.3 HIV RNA and DNA measurement

We measured HIV-1 RNA levels in plasma, TFS eluates and CVL fluid using the Abbott RealTime HIV-1 assay (Abbott Laboratories, Abbott Park, IL, USA) at visits 1–2 and 4–7. TFS samples were eluted in 0.9 ml Abbott DBS Elution Buffer prior to the assay. For all sample types, the limit of quantitation was 40 copies/ml without dilution. HIV RNA viral loads for TFS were not adjusted for elution volume and so were considered cp/ml of eluate. When repeat testing was performed, TFS eluates were diluted 1:5, and the limit of quantification was then 200 cp/ml.

DNA was extracted from frozen CVL cell pellets using a proprietary M-PVA Magnetic Bead Technology on a Chemagic MSM I robotic system (Perkin Elmer, Baesweiler, Germany) by the UNC BioSpecimen Processing Facility. Cell pellets were thawed and resuspended in 1 ml Tissue Lysis Buffer (Perkin Elmer), and the lysate was added to the MSM I deep-well block containing 60 μg RNase A (Qiagen) and incubated for 20 min at ambient temperature. Fifteen microliters of Proteinase K (Perkin Elmer) was mixed into the samples and incubated for 2 h at 55°C with moderate shaking, followed by elution with 170 μl buffer. Eluates were spun for 6 min at 14,000×*g* to remove residual beads. Optical densities were obtained (Thermo Scientific Nanodrop), after which the DNA was stored at −80°C. HIV-1 DNA was detected using Droplet Digital PCR (ddPCR) using HIV primers and probe designed by Palmer et al. [24] with Bio-Rad iScript and ddPCR Supermix for Probes. Samples with low droplet count were digested with *Msc*I before repeat testing. The lower limit of detection is approximately 1 cp/reaction.

### 2.4 Statistical analysis

Since most sub-Saharan African countries (including Malawi) now recommend that all women be initiated on ART regardless of CD4+ T cell count, we restricted all analyses to women who were taking ART (by self-report) at the time of enrollment to mimic current real-world conditions. We compared the frequency and magnitude of genital HIV shedding (measured by HIV RNA viral load in CVL and TFS) between the two study visits before and the four study visits after randomization/starting contraception, and between the two contraceptive arms using multivariable repeated measurements models fit by generalized estimating equations. We evaluated the interaction between contraceptive initiation and arm in each model using a Wald test. HIV RNA viral load results were log_10_ transformed for analysis, and for samples where HIV RNA was not detected by the assay, a value of half the lowest detected value was assigned. The multivariable models were adjusted for baseline plasma HIV RNA viral load and CD4+ T cell count. We conducted all analyses using SAS 9.3 (SAS Institute, Cary, NC, USA).

### 2.5 Ethical considerations

The study was approved by the University of North Carolina Institutional Review Board (IRB), the Malawi National Health Sciences Research Committee, the Malawi Pharmacy Medicines and Poisons Board and the IRB of the U.S. Centers for Disease Control and Prevention.

## 3 Results

**Fig. 1:**
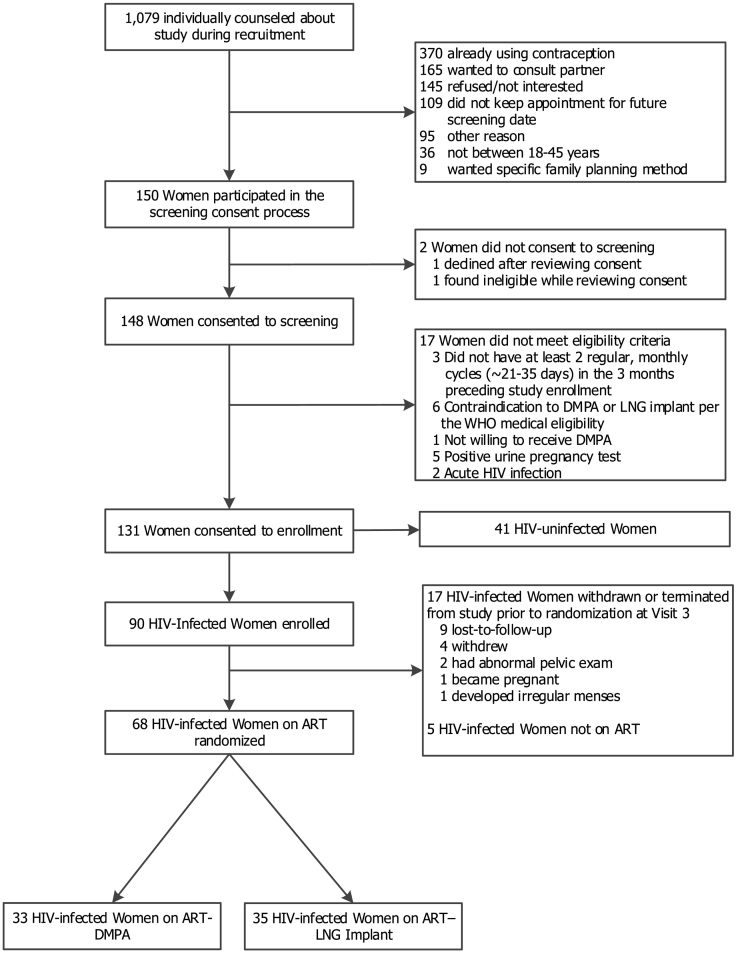
Flowchart for study recruitment, screening, enrollment, and randomization.

During our recruitment period from April 01, 2014, to December 31, 2014, study nurses counseled and prescreened 1079 women for our study (Fig. 1). Of these women, we enrolled 90 HIV-infected women. We terminated or withdrew 17 women from the study prior to randomization and randomized 73 HIV-infected women (37 to DMPA and 36 to LNG implant). We excluded 5 women because they were not taking ART at the time of enrollment (4 randomized to DMPA, 1 randomized to the LNG implant), giving us a total of 68 women (33 randomized to DMPA, 35 randomized to LNG implant) for this analysis.

The median age of the 68 women was 36 years (IQR 30.0–39.0) in the DMPA arm and 34 years (IQR 29.0–39.0) in the LNG implant arm (Table 1). Over 80% of women in each study arm had been receiving ART (mostly efavirenz-based regimens) for at least 1 year. Their median CD4+ T cell count at enrollment was 406.0 cells/mm^3^ (IQR 270.0–501.0) in the DMPA arm and 307.0 cells/mm^3^ (IQR 207.0–446.0) in the LNG implant arm. Plasma HIV viral load at enrollment was below the limit of quantification (<40 copies/ml) in 81.8% (*n*=27) of women in the DMPA arm and 71.4% (*n*=25) in the LNG implant arm.

**Table 1:** Baseline characteristics for 68 HIV-infected women on antiretroviral therapy enrolled and randomized in the study, Lilongwe, Malawi

Characteristics at study enrollment	DMPA (*n*=33) Study arm		LNG implant (*n*=35) Study arm	
	N	%	N	%
Marital status				
Married	21	63.6	22	62.9
Separated/divorced/widowed	12	36.4	13	37.1
Never married				
Education				
<Primary	18	54.6	13	37.1
Primary	4	12.1	8	22.9
Some secondary	11	33.3	14	40.0
Any past use of hormonal contraception				
Yes	24	72.7	27	77.1
No	9	27.3	8	22.9
Any sexual partners in the past 3 months				
Yes	21	63.6	23	65.7
No	12	36.4	12	34.3
Diagnosed with HIV				
≤1 year ago	2	6.1	5	14.3
1–5 years ago	22	66.7	15	42.9
>5 years ago	9	27.3	15	42.9
Started on antiretroviral therapy				
≤1 year ago	4	12.1	6	17.1
>1 year ago	29	87.9	29	82.9
Currently taking efavirenz				
Yes	28	84.9	30	85.7
No, nevirapine	5	15.2	3	8.6
No, atazanavir/ritonavir	0	0.0	2	5.7
Plasma HIV RNA viral load (copies/ml)^a^				
<40	27	81.8	25	71.4
40–999	2	6.1	3	8.6
1000–4999	0	0.0	3	8.6
≥5000	4	12.1	4	11.4

	Median	IQR	Median	IQR

Age (years)	36.0	30.0–39.0	34.0	29.0–39.0
Number of living children	3.0	2.0–4.0	3.0	2.0–3.0
Weight (kg)^b^	53.3	49.9–57.9	57.2	53.0–64.4
CD4+ T cell count^b^	406.0	270.0–501.0	307.0	207.0–446.0

a Measured at follicular phase.

b Weight and CD4+ T cell count were all measured at follicular phase.

**Fig. 2:**
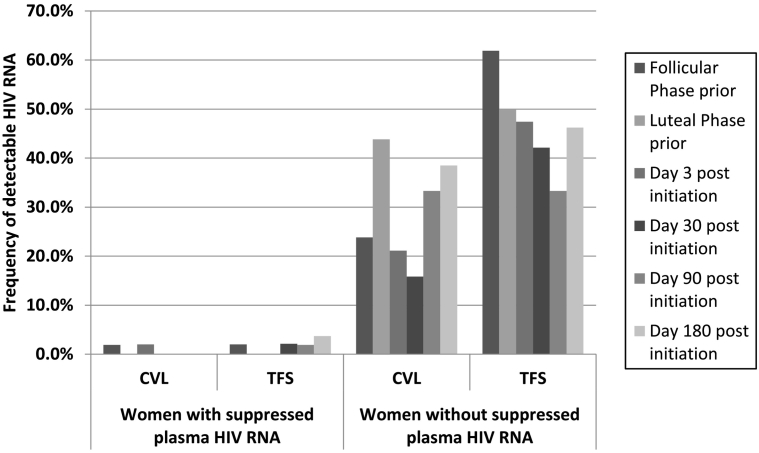
Frequency of HIV RNA detected in CVL fluid (≥40 cp/ml) or TFS (≥200 cp/ml) in samples from women with HIV RNA detected or not in the plasma at the time of specimen collection. The first two visits were prior to randomization/contraceptive initiation, and the last four visits were between 3 and 180 days post initiation of contraception.

HIV RNA levels were higher in plasma samples than genital samples and higher in TFS than in CVL fluid (Table 2). Likewise, HIV RNA was detectable more frequently in TFS compared with CVL (Fig. 2). Genital HIV RNA was detectable more often among women who had detectable HIV in their plasma at the time of specimen collection (Fig. 2) and had higher plasma HIV RNA levels (not shown). Overall, there was little HIV detected in genital secretions when the plasma VL was undetectable.

**Table 2:** Plasma HIV RNA viral load and genital HIV RNA viral load upper percentiles (copies/ml), CVL vs. TFS, for 68 HIV-infected women currently taking antiretroviral medications at enrollment in the study

	Plasma								Genital			
						CVL fluid				TFS		
	*N*<40 copies/ml	Percentile			*N*<40 copies/ml	Percentile			*N*<40 copies/ml	Percentile		
Study visit		90th	95th	Maximum		90th	95th	Maximum		90th	95th	Maximum
Follicular phase prior to contraceptive initiation	52	6342	20,645	223,582	64	<40	44	1352	57	1104	1879	5757
Luteal phase prior to contraceptive initiation	48	6842	22,597	180,902	56	<40	127	1471	55	<200	3855	5290
Day 3 post initiation of contraceptive method	53	6278	14,791	136,174	59	<40	196	927	58	508	920	13,148
Day 30 post initiation	52	6373	9794	92,774	59	<40	<40	3478	55	674	2441	9550
Day 90 post initiation	54	676	6877	83,548	56	<40	560	1544	56	<200	282	9734
Day 180 post initiation	58	2601	6088	131,948	63	<40	<40	1104	57	<200	1387	10,210

At visit 1, 5.7% (*n*=2) of LNG implant users and 6.1% (*n*=2) of DMPA users had at least 40 cp/ml of HIV RNA in their CVL (Table 3). With TFS, 12.1% (*n*=4) of the DMPA users and 17.7% (*n*=6) of the LNG implant users had detectable HIV RNA (at least 200 cp/ml) at enrollment. There was no significant difference in HIV RNA detection in CVL before and after contraceptive initiation in either the DMPA arm (RR=0.82 [0.20–3.37]) or the LNG implant arm (RR=1.31 [0.43–4.03]), adjusted for baseline plasma HIV RNA viral load and CD4+ T cell count. The interaction between contraceptive initiation and study arm was not significant (p=.60). Among the TFS samples from the same women, the effect of contraceptive initiation varied by study arm (p value for interaction=.01); higher detectable genital HIV RNA before, compared with after, contraceptive initiation was found in the LNG implant arm (RR=0.40 [0.18–0.85]) but not in the DMPA arm (RR=1.37 [0.81–2.33]) after adjustment for baseline plasma HIV RNA viral load and CD4+ T cell count. The magnitude of genital HIV RNA did not differ after contraceptive initiation or by study arm in either CVL or TFS (Table 3).

**Table 3:** Genital HIV RNA viral load by study visit and study arm among 68 women on antiretroviral therapy, Lilongwe, Malawi

	CVL fluid		TFS	
	DMPA (*n*=33)	LNG implant (*n*=35)	DMPA (*n*=33)	LNG implant (*n*=34)
Study visit	#≥40 copies/*N* (%)	#≥40 copies/*N* (%)	#≥200 copies/*N* (%)	#≥200 copies/*N* (%)
Follicular phase prior to contraceptive initiation	2/33 (6.1%)	2/35 (5.7%)	4/33 (12.1%)	6/34 (17.7%)
Luteal phase prior to contraceptive initiation	2/28 (7.1%)	1/31 (3.2%)	3/28 (10.7%)	1/31 (3.2%)
Day 3 post initiation of contraceptive method	3/31 (9.7%)	2/33 (6.1%)	3/32 (9.4%)	4/33 (12.1%)
Day 30 post initiation	1/27 (3.7%)	1/34 (2.9%)	4/28 (14.3%)	3/34 (8.8%)
Day 90 post initiation	1/27 (3.7%)	2/32 (6.3%)	2/27 (7.4%)	1/32 (3.1%)
Day 180 post initiation	0/33 (0%)	3/33 (9.1%)	3/29 (10.3%)	2/33 (6.1%)

Quantitative genital HIV RNA viral load (log10 copies/ml)	Regression coefficient (95% CI)^a^		Regression coefficient (95% CI)^a^	

Before vs. after progestin contraception initiation:				
DMPA	−0.05 (−0.15 to 0.05)		−0.04 (−0.19 to 0.11)	
LNG implant	−0.01 (−0.16 to 0.13)		0.14 (−0.02 to 0.31)	

Detectable genital HIV RNA viral load	RR (95% CI)^a^		RR (95% CI)^a^	

Before vs. after progestin contraception initiation:				
DMPA	1.22 (0.30–4.98)		0.73 (0.43–1.24)	
LNG implant	0.76 (0.25–2.35)		2.53 (1.18–5.41)	

a Results from a multivariable regression model fit using generalized estimating equations including an interaction term for contraception initiation by study arm, and adjusted for baseline plasma HIV RNA viral load and CD4+ T cell count.

HIV DNA was detected in only 4 CVL cell samples of 360 samples tested: 3 prior to contraceptive initiation and 1 after LNG implant initiation. The woman with HIV DNA detected after LNG implant initiation (visit 5) also had HIV DNA detected prior to contraceptive initiation (visit 1), and HIV RNA was detectable in both the CVL fluid and TFS samples at both of these visits. For the other two women with detectable HIV in CVL fluid prior to contraceptive initiation, HIV RNA was not found to be detectable in either the CVL or TFS samples at those same visits.

## 4 Discussion

HIV-infected women receiving ART in our study had low rates of genital HIV shedding before and after initiation of progestin-only contraception. There was a significantly higher detection rate of HIV RNA in TFS before starting the LNG implant than after starting it, which was not seen with DMPA. In both the LNG implant arm and the DMPA arm, there was no increase in genital HIV shedding after contraception initiation. Consistent with other studies, we also found higher HIV RNA levels in TFS samples than in CVL fluid samples [17], [18], likely due to TFS being undiluted at the time of collection.

The frequency of genital shedding was low when plasma HIV viral load was undetectable, appearing in less than 4% of the 68 participants, regardless of contraceptive use. This low frequency of shedding is consistent with a low risk of transmission in HIV-infected women taking ART [7]. This finding is reassuring since DMPA and the LNG implant are two commonly used contraceptives methods by HIV-infected women in sub-Saharan Africa [2]. These results also add to the very limited HIV genital shedding information available for the LNG implant.

The results of our study are consistent with the results of two recent studies that assessed HIV shedding among women initiating ART [11], [12], [25]. A prospective cohort study of 188 sex workers initiating ART in Burkina Faso evaluated plasma and CVL HIV-1 RNA every 3–6 months for up to 8 years [11]. The study found that neither DMPA (adjusted OR 1.32, CI 0.42–4.16) nor oral contraceptive use (adjusted OR 1.57, CI 0.75–3.27) was associated with increased CVL HIV RNA when adjusted for plasma viral load. The second study, a prospective cohort of 102 HIV-infected women initiating ART in Kenya, also found no association between DMPA use and qualitative detection of cervical HIV RNA (adjusted OR 1.41, CI 0.54–3.67) or plasma HIV RNA (adjusted OR 0.81, CI 0.47–1.39) [12]. The results of our study are also consistent with other studies of women taking ART, which have found cervical HIV RNA detectable in only 3%–33% of women studied, with much lower probability of shedding when the plasma HIV is suppressed [8], [22], [26], [27], [28]. Detectable HIV load in the plasma can be a result of suboptimal response to ART or imperfect ART adherence. Of note, plasma HIV RNA was quantifiable in almost a quarter of our study participants at baseline, further highlighting “real-world” issues with adherence to ART and risks of HIV transmission to partners.

Our study has some limitations. The sample size is relatively small, and quantitative differences in genital HIV RNA levels were difficult to assess due to the low frequency of detectable HIV RNA in women taking ART. However, using the observed frequency of detectable genital HIV RNA, the study had 80% power to detect a difference in the frequency of detectable genital HIV RNA of 8.7% (RR=2.58) in CVL fluid and 10.9% (RR=1.98) in TFS after initiation of hormonal contraception. Also, we relied on self-report for contraceptive nonuse in the 6 months prior to enrollment, which may be subject to misreporting. However, our study has the major methodologic strength of being a randomized trial and therefore is much less subject to confounding. To our knowledge, this is the only randomized trial comparing the effect of two contraceptive methods on genital HIV shedding. Contraceptive methods were administered by study staff, thus avoiding ascertainment bias that may occur with self-reported contraceptive use, and the time of contraceptive administration was known. As contraception was provided through the study, adherence was ascertained at each study visit. Furthermore, we measured HIV genital shedding from two specimen types at multiple time points before and after initiation of LNG implant and DMPA, which were timed to correspond with important points in the pharmacokinetic curve of the contraceptive hormones and allowed for a direct comparison of the two specimen types.

In conclusion, our study showed that initiation of DMPA or the LNG implant was not associated with increased genital HIV shedding during the first 6 months of use among women taking ART. These findings are consistent with growing evidence that progestin contraception is not associated with increased HIV transmission risk in the context of ART [11], [12].
